# Genomic prediction within and across maize landrace derived populations using haplotypes

**DOI:** 10.3389/fpls.2024.1351466

**Published:** 2024-03-22

**Authors:** Yan-Cheng Lin, Manfred Mayer, Daniel Valle Torres, Torsten Pook, Armin C. Hölker, Thomas Presterl, Milena Ouzunova, Chris-Carolin Schön

**Affiliations:** ^1^Chair of Plant Breeding, TUM School of Life Sciences, Technical University of Munich, Freising, Germany; ^2^Bayer CropScience Deutschland GmbH, Borken, Germany; ^3^Sugar Beet Breeding, Strube Research GmbH & Co. KG, Söllingen, Germany; ^4^Animal Breeding and Genomics, Wageningen University & Research, Wageningen, Netherlands; ^5^Product Development Maize and Oil Crops, KWS SAAT SE & Co. KGaA, Einbeck, Germany

**Keywords:** haplotype construction, genomic prediction, across population prediction, parameter tuning, landraces

## Abstract

Genomic prediction (GP) using haplotypes is considered advantageous compared to GP solely reliant on single nucleotide polymorphisms (SNPs), owing to haplotypes’ enhanced ability to capture ancestral information and their higher linkage disequilibrium with quantitative trait loci (QTL). Many empirical studies supported the advantages of haplotype-based GP over SNP-based approaches. Nevertheless, the performance of haplotype-based GP can vary significantly depending on multiple factors, including the traits being studied, the genetic structure of the population under investigation, and the particular method employed for haplotype construction. In this study, we compared haplotype and SNP based prediction accuracies in four populations derived from European maize landraces. Populations comprised either doubled haploid lines (DH) derived directly from landraces, or gamete capture lines (GC) derived from crosses of the landraces with an inbred line. For two different landraces, both types of populations were generated, genotyped with 600k SNPs and phenotyped as lines per se for five traits. Our study explores three prediction scenarios: (i) within each of the four populations, (ii) across DH and GC populations from the same landrace, and (iii) across landraces using either DH or GC populations. Three haplotype construction methods were evaluated: 1. fixed-window blocks (FixedHB), 2. LD-based blocks (HaploView), and 3. IBD-based blocks (HaploBlocker). In within population predictions, FixedHB and HaploView methods performed as well as or slightly better than SNPs for all traits. HaploBlocker improved accuracy for certain traits but exhibited inferior performance for others. In prediction across populations, the parameter setting from HaploBlocker which controls the construction of shared haplotypes between populations played a crucial role for obtaining optimal results. When predicting across landraces, accuracies were low for both, SNP and haplotype approaches, but for specific traits substantial improvement was observed with HaploBlocker. This study provides recommendations for optimal haplotype construction and identifies relevant parameters for constructing haplotypes in the context of genomic prediction.

## 1 Introduction

High-density marker technologies have provided researchers with the opportunity to harness the power of haplotypes. In genetics, haplotypes refer to the combination of alleles at multiple loci on the same chromosomal homolog (Griffiths et al., 2010). Hence, haplotypes allow for a more comprehensive representation of genetic variation in comparison to single nucleotide polymorphisms (SNPs). They can capture ancestral information and identify rare alleles in the population under study (Bhat et al., 2021). Haplotypes are more likely to exhibit linkage disequilibrium (LD) with causal polymorphisms of QTL than individual SNPs (Balding, 2006) and should capture information on local epistasis (Jiang et al., 2018). In addition, the use of haplotypes can somewhat mitigate the “large p, small n problem” of linear models (Pattaro et al., 2008), unless populations under study exhibit high allelic diversity leading to high number of haplotype variants. These characteristics are assumed to make haplotypes advantageous over individual SNPs in many applications of genomic research, including genome-wide association studies (Mayer et al., 2020) and genomic prediction (Hess et al., 2017).

In their seminal paper on genomic prediction, Meuwissen et al. (2001) assumed the QTL alleles to be in LD with haplotypes constructed from two markers. Various studies have since investigated the use of haplotypes for genomic prediction, both in simulations and in experimental studies, in different species, diverse datasets, for different traits and for different prediction methods. A comprehensive overview of studies from plant breeding is given in Difabachew et al. (2023). In general, most studies confirmed the assumption that employing haplotypes for genomic prediction of genetic values can be advantageous over SNP-based approaches, but the performance of haplotype-based prediction depended on various factors, including the traits under consideration, the genetic structure of the population under study and in particular the specific method used for haplotype construction (Sallam et al., 2020; Won et al., 2020; Ye et al., 2022; Difabachew et al., 2023; Weber et al., 2023).

Haplotype construction methods differ in their treatment of LD and relatedness, which in turn are a function of the genetic structure of the population. Thus, for the same species the same method can lead to different haplotype structures in different populations. Fixed-window methods create haplotypes based on genomic regions spanning a fixed number of adjacent SNPs without considering LD or relatedness in the population (Cuyabano et al., 2015; Hess et al., 2017; Sallam et al., 2020). LD based methods form variable-length haplotype blocks along the genome based on the LD structure prevalent in the population (Gabriel et al., 2002; Barrett et al., 2005). The HaploBlocker method defines haplotypes by considering group-wise identity-by-descent (IBD) chromosome segments among predefined subgroups in the population (Pook et al., 2019). This approach could be beneficial when the sub-populations in the dataset are clearly defined. To investigate the interaction between construction method and population structure we assessed the prediction accuracy of haplotype versus SNP-based prediction with three different haplotype construction methods in each of three prediction scenarios differing with respect to the genomic structure of the training and the prediction data sets.

The basis for the three prediction scenarios was the phenotypic and genotypic data of four maize landrace derived populations generated by two different breeding approaches. Two populations comprise doubled-haploid (DH) lines generated directly from S_0_ plants of two different landraces, the other two populations were derived from the same two landraces but by crossing landrace S_0_ plants with a common inbred line and subsequent selfing (Hölker et al., 2019; Hölker et al., 2022). Differences in genetic diversity, LD patterns, and levels of relatedness within and among these populations make the dataset ideal for the investigation of haplotype-based genomic prediction within populations, across populations from the same landrace and across landraces.

Objectives of our study were to (i) assess the accuracy of haplotype- and SNP-based genomic prediction for five agronomic traits in landrace derived maize populations, (ii) compare haplotype construction methods with respect to their prediction accuracies in different prediction scenarios, and (iii) investigate impacts of parameter settings conditional on the prediction scenario.

## 2 Materials and methods

### 2.1 Data set

#### 2.1.1 Plant material

We used four different maize populations developed from the two European flint maize landraces, Kemater Landmais Gelb (KE) and Petkuser Ferdinand Rot (PE) (Hölker et al., 2019; Mayer et al., 2020). From each landrace, a doubled haploid (DH) population was derived directly from S_0_ plants, and a gamete capture (GC) population of S_1:2_ plants was generated by crossing S_0_ plants with the capture line, FV2, and subsequent selfing (Hölker et al., 2022). The entire dataset comprises 1,417 landrace derived lines (DH_KE = 471, DH_PE = 402, GC_KE = 274, GC_PE = 270).

#### 2.1.2 Phenotypic data

Field design and trait assessment were described in detail by Hölker et al. (2019) and Hölker et al. (2022). In brief, the DH lines and GC-S_1:2_ lines were evaluated at two different locations, Roggenstein (ROG) and Einbeck (EIN), in Germany in two years (2017 and 2018). Line *per se* performance was assessed for five traits, early vigor at V6 stage (EV_V6, 1-9 score), plant height at V6 stage (PH_V6, cm), final plant height (PH_final, cm), female flowering time (DtSILK, days from sowing to 50% of plants in a plot silked) and severity of root lodging at R6 stage (RL_R6, 1-9 score). For all traits, we calculated adjusted means across the four environments as described in Hölker et al. (2019).

#### 2.1.3 Genotypic data

All DH lines and GC-S_1_ plants were genotyped with the 600k Affymetrix Axiom Maize Array (Unterseer et al., 2014). Quality filtering and imputation followed Hölker et al. (2019) for DH populations and Hölker et al. (2022) for GC populations. Only markers of the best quality class (Unterseer et al., 2014) were selected. Subsequently, markers with ambiguous physical positions on the B73 reference genome AGPv4 (Jiao et al., 2017) and markers and individuals with >10% missing rate were removed. In DH populations, markers and individuals with >5% heterozygosity were discarded. The remaining heterozygous genotype calls were set as missing values. Imputation and phasing were performed separately in each population. Missing genotype calls in DH populations were imputed with Beagle version 5.0 (Browning et al., 2018). Imputation and gamete phasing of GC-S_1_ lines were done using Beagle version 5.0, with parameters iteration = 50, phase-segment = 10, and phase-states = 500. After all filtering steps, a total of 486,971 polymorphic SNPs remained and were used for further analysis.

### 2.2 Prediction scenarios

**Figure 1:**
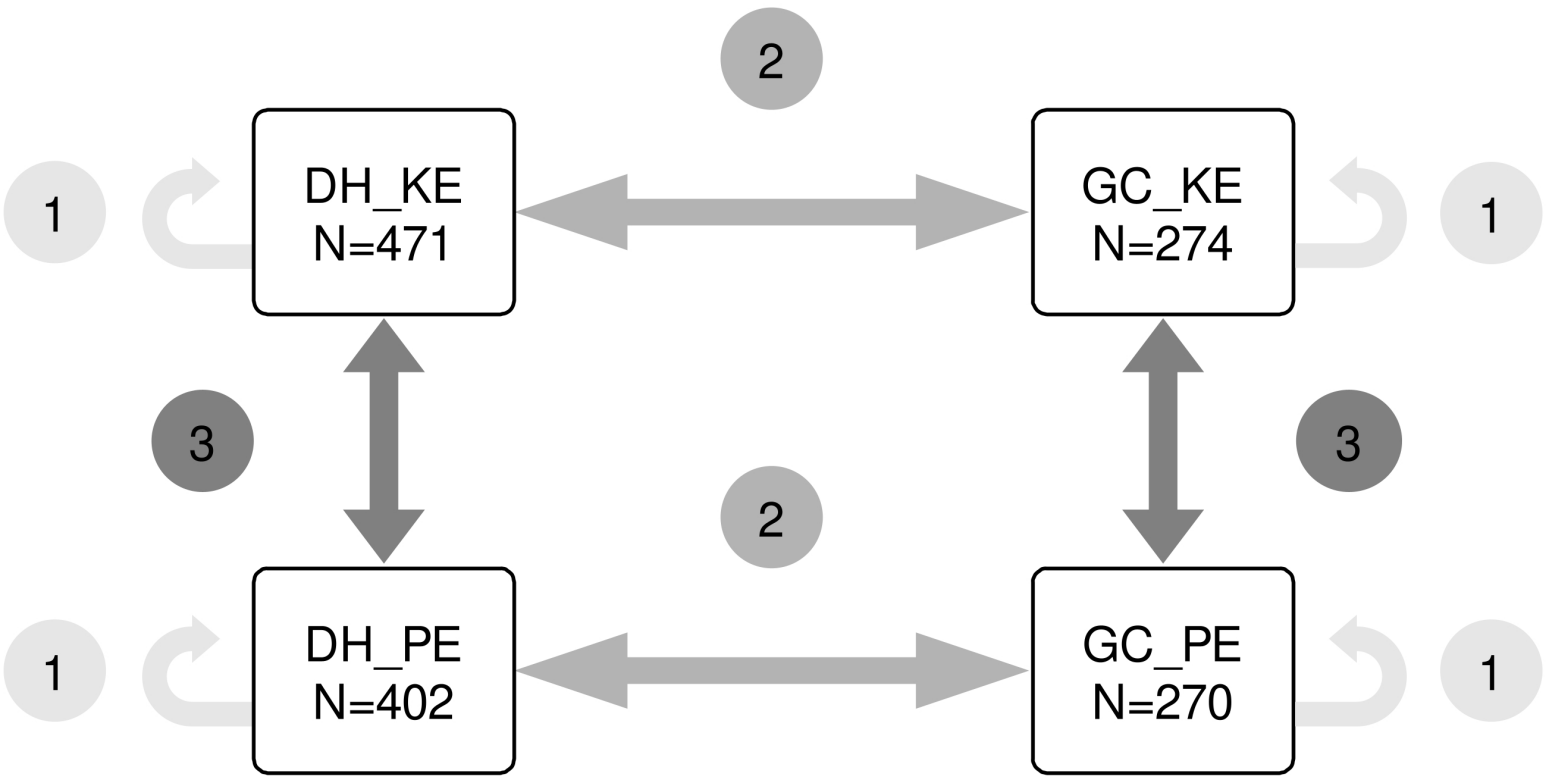
Scenarios of genomic prediction. Scenario 1 is prediction within population. Scenario 2 is prediction across DH and GC populations of the same landrace. Scenario 3 is prediction across landraces with a focus on populations that use the same breeding approach (DH or GC).

Three prediction scenarios were devised (illustrated in **Figure 1**). In scenario 1 (within population prediction) the training set (TS) and the prediction set (PS) originated from the same population. In scenario 2 (across population prediction) the TS and PS were of different population type but from the same landrace. In scenario 3 (across landrace prediction) TS and PS were of the same population type but from different landraces. In scenario 1, all lines of a given population were used for ten iterations of five-fold cross-validation. In scenario 2 and 3, the sample size for the training set was restricted to 270 lines by the smallest population, GC_PE. To align sample sizes with the five-fold cross-validation approach in scenario 1, 200 lines from one population were randomly sampled as TS, and 50 lines from the other population were sampled as PS. This sampling procedure was repeated 100 times. Prediction accuracy was calculated as the Pearson correlation between predicted genetic values and observed phenotypic values divided by the square root of the heritability, *h^2^*, of the prediction set (Dekkers, 2007).

### 2.3 Haplotype construction methods

Three haplotype construction methods were implemented in this study. The first two were the fixed-window and LD-based method, where haplotype blocks (loci), consisting of groups of SNP markers, were defined either according to their number and order on the physical map or based on the LD between the markers, respectively. Haplotype alleles were then defined as combinations of gamete SNP alleles within these blocks. The third method used in this study was an IBD-based method suggested by Pook et al. (2019). This method generates haplotype alleles of arbitrary length without specifying haplotype loci. In scenario 1, haplotypes were constructed separately for each of the four populations. In scenario 2 and 3, haplotypes were constructed using merged genotypic data of the TS and PS.

#### 2.3.1 Fixed-window method (FixedHB)

Haplotype blocks were created using a fixed number of adjacent SNPs, forming non-overlapping blocks from the first to the last SNP at the end of a chromosome on the physical map. To explore the impact of block size on prediction accuracy, five different block sizes (5, 10, 20, 50 and 100 SNPs) were tested.

#### 2.3.2 LD-based method (HaploView)

We utilized the software HaploView (Barrett et al., 2005) which offers three different algorithms for clustering SNPs based on their LD. The first algorithm, GAB, employs the measurement of *D’*, the coefficient of linkage disequilibrium *D* normalized by the maximum value, to define SNP pairs in LD. SNP pairs were in “strong LD” if the 95% confidence interval of *D’* had an upper bound above 0.98 and a lower bound above 0.7 (default threshold). A block would be formed if 95% of the SNP pairs exhibit LD (Gabriel et al., 2002). The second algorithm uses the four-gamete rule [4GAM; (Wang et al., 2002)] and defines a SNP pair to be in LD if one of the four possible two-marker haplotypes has a frequency below 0.01. Blocks are formed by consecutive markers in LD. The third algorithm, solid spine of LD (SPINE), uses the same measurement as GAB (*D’*) for defining SNP pairs in LD. If the first and the last SNP are in LD, all the intermediate SNPs will be included in a block. Parameter settings for each algorithm were kept at their default values given by HaploView. To preserve the gamete phase information of double heterozygotes in GC lines, the two phased gametes of a GC individual were treated as two pseudo-inbred individuals.

#### 2.3.3 IBD-based method (HaploBlocker)

To construct a haplotype library, the R-package HaploBlocker (Pook et al., 2019) was used. With HaploBlocker, a set of haplotypes is defined that captures a large proportion of the genetic variation with a limited number of haplotypes. The program allows the identification of haplotypes with a pre-defined minimum frequency, which enables the identification of genomic segments identical-by-descent (IBD) across populations. Four essential parameters of the haplotype library were tested to investigate their impact on haplotype library construction.

##### 2.3.3.1 Window size

The *window size* parameter determines the number of SNPs that form the initial window at the beginning of cluster-building. Its value will affect the length of haplotypes in the final haplotype library. Five different values (5, 10, 20 (default), 50 and 100) were used in this study. Additionally, a special mode (*multi_window_mode*) allowing multiple window sizes (5, 10, 20, and 50) simultaneously was also evaluated.

##### 2.3.3.2 Minimum number of cells as the most relevant block

The *MCMB* (parameter *min_majorblock* in R/HaploBlocker) plays a crucial role in the haplotype filtering process. It serves as a control to achieve a balance between information conservation (genome coverage) and reduction of variants (total number of haplotypes). Lower values result in more haplotypes and higher genome coverage. The default value is 5000, and six different values (1, 500, 1250, 5000, 20000 and 80000) were tested in this study.

##### 2.3.3.3 Target coverage

To achieve the desired genome coverage, i.e. proportion of genome covered by at least one haplotype, the HaploBlocker program will automatically adapt the *MCMB* value for haplotype library construction. *Target coverage* values of 80, 85, 90, 95, and 99 were tested. If *target coverage* is not selected, the program will construct the haplotypes only with the designated *MCMB* value. For all haplotype libraries, with or without setting of *target coverage*, genome coverage was calculated after haplotype construction for further comparison.

##### 2.3.3.4 Minimum occurrence of a haplotype in subgroup

By setting a minimum occurrence threshold for each haplotype in pre-defined subgroups, e.g. the TS and PS in scenarios 2 and 3, we can exclude haplotypes with very low frequency in either subgroup and retain only the common shared ones. Six different values (0, 5, 20, 40, 80, and 160) were tested to assess their impact on the resulting haplotype library. To identify haplotypes from the capture line, FV2, its genotypic data was included in the GC population during haplotype construction in scenario 2.

*Window size* and *target coverage* were tuned in within population prediction (scenario 1), while *MCMB* and *Min Subgroup* were evaluated for their impact on across population prediction (scenario 2 and 3). Due to the high genotyping accuracy of the SNP array data, error control when building the haplotype library was set to 0 (parameter *merging_error* in R/HaploBlocker). For all other parameters which were not explicitly mentioned default values were used.

### 2.4 Genomic prediction model

We employed genomic best linear unbiased prediction (GBLUP) following Hölker et al. (2022). In the GBLUP model:


 y=1μ+Zu+e

y is a vector of adjusted means averaged across environments of the training set, 1 is a vector of ones, μ is the overall mean, Z is an incidence matrix. u is a vector of random genetic effects with the distribution $u\;∼\;N\left(0,\;U\sigma_{g}^{2}\right)$ , U is the realized relationship matrix calculated on the basis of genotypic data, and $\sigma_{g}^{2}$ is the genetic variance pertaining to the GBLUP model. e is a vector of residuals assumed to be independent and identically distributed with $e\;∼\;N\left(0,\;I\sigma_{e}^{2}\right)$ , where I is the identity matrix, and $\sigma_{e}^{2}$ is the residual variance pertaining to the GBLUP model. For SNP-based genomic prediction the matrix $U_{S}$ was the genomic relationship matrix (GRM) calculated by VanRaden method 1 (VanRaden, 2008).

For haplotype-based genomic prediction, the relationship matrix $U_{H}$ , was calculated using haplotype matrix $M_{nxp}$ ( n = number of genotypes, p = total number of haploblock alleles). Each haplotype allele was treated as a pseudo-marker, coded as 0, 1 or 2 representing the count of the haplotype carried by an individual. Haplotype alleles present in only one individual in a defined population were excluded from the analysis. Estimation of variance components and the GBLUP model were implemented using R/ASReml 4.1 (Gilmour et al., 2015).

Genomic relationship matrices built on SNPs and different haplotype construction methods were compared with the Mantel test (Mantel, 1967). The Mantel test assesses the correlation between the *c*(*c*−1)/2 entries below the diagonal of two symmetric *c*×*c*-dimensional matrices. The significance of the correlation is evaluated by permuting columns and rows of the first matrix while keeping the second matrix fixed. We conducted the Mantel test by using *mantel()* in R/vegan (Oksanen, 2010) with default settings. Differences in prediction accuracies between haplotype-based and SNP-based methods were tested with a Wilcoxon signed-rank test with Bonferroni correction for multiple testing using function *wilcox.test* and *p.adjust* in R (R Core Team, 2022).

### 2.5 FV2 haplotype composition in scenario 2

We hypothesized that the haplotypes shared between FV2 and the DH population had a negative effect on prediction accuracy. These haplotypes might be alike in state but might have different QTL effects in DH and GC. To assess the influence, we quantified the prevalence of FV2 haplotypes in the DH population. We calculated the ratio of FV2 haplotype alleles found in the respective DH population divided by the total number of haplotype alleles present. We minimized the number of FV2 haplotypes to maximize the overlap of haplotypes between DH and GC populations, tuning the *Min Subgroup* parameter from 10 to 160 under the default *MCMB* value 5000.

## 3 Results

### 3.1 Characteristics of haplotypes using three different construction methods

**Figure 2:**
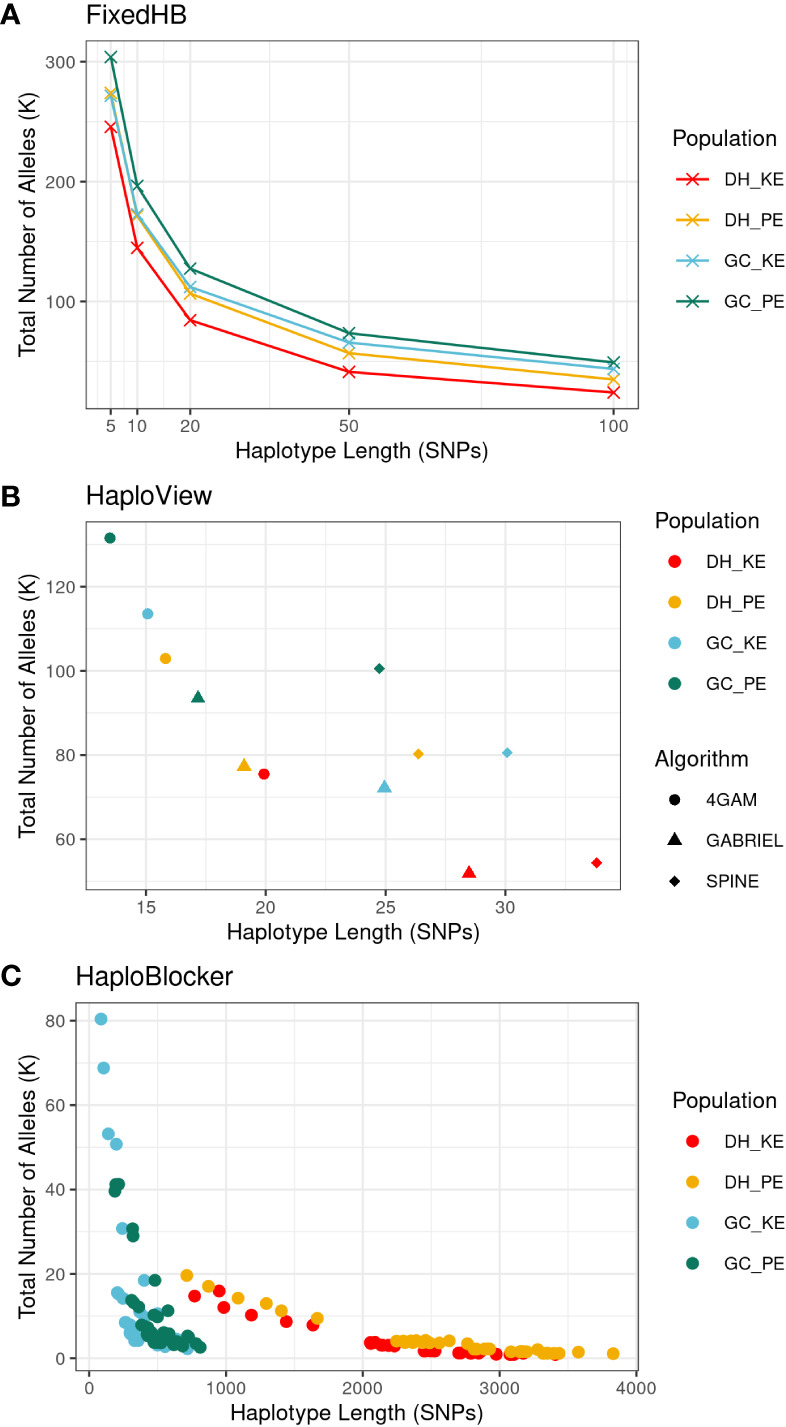
Characteristics of haplotypes generated from three methods, FixedHB **(A)**, HaploView **(B)** and HaploBlocker **(C)**, with different parameter settings in scenario 1. Each data point corresponds to a distinct haplotype set, with the x-axis indicating the averaged haplotype length and the y-axis representing the total number of haplotype alleles. Colors differentiate the four populations. For HaploView, symbols indicate the algorithm used. HaploBlocker parameter settings are not distinguished to maintain clarity.

**Figure 2** provides an overview of the characteristics of haplotypes generated by three different construction methods. In the FixedHB method, haplotype length was predetermined and fixed, in general, resulting in the highest total haplotype allele number among the three methods. As the haplotype length increased, particularly in the range of 5 to 50 SNPs, the total allele number decreased due to the presence of LD between adjacent SNPs. The HaploView methods produced haplotype blocks ranging from 14 to 34 SNPs in average length. The 4GAM method generated the shortest haplotype blocks, while the SPINE method produced the longest haplotype blocks. For the HaploBlocker method, the length and total number of haplotypes varied significantly depending on the specific settings used. Both *window size* and *target coverage* parameters had an impact on haplotype length, with *target coverage* showing a particularly strong effect in DH populations (**Supplementary Figure S1**). Generally, HaploBlocker generated the fewest and longest haplotypes compared to the other methods. It also displayed a clear differentiation in haplotype structures between DH and GC populations. The GC populations exhibited a higher total number of haplotype alleles compared to the DH population, which can be attributed to the introduction of new haplotype alleles from FV2 and the occurrence of new recombination events in the GC populations. Longer haplotypes were observed in the DH populations. The presence of quite long haplotypes with low allele frequencies suggested some large identical-by-descent (IBD) segments between DH lines. These segments were most likely broken up by recombination when GC lines were produced and by potential phasing errors during GC imputation (**Figure 2**).

**Table 1** compares the genomic relationship matrix (GRM) derived from SNPs and GRMs constructed with haplotypes using the three methods with optimal parameter settings (described in the following section). All haplotype-based GRMs were significantly correlated with the SNP-based GRM in the Mantel test (p < 0.001), with the HaploBlocker method showing the lowest Mantel correlation with the SNP-based GRM. **Table 2** shows the reduction in explanatory variables for the three haplotype methods compared to the number of SNPs. The strongest reduction was observed for the HaploBlocker method with the number of haplotypes being only 3-15% of the number of SNPs. Despite this strong reduction, the HaploBlocker GRM maintained a high correlation (> 0.9) with the SNP-based GRM.

**Table 1:** Comparison of SNP-based and haplotype-based genomic relationship matrices (GRM).

	FixedHB (window size: 20)	HaploView (GAB)	HaploBlocker (window size: 20, target coverage: 99%)
DH_KE	0.996	0.980	0.930
DH_PE	0.996	0.980	0.941
GC_KE	0.992	0.966	0.944
GC_PE	0.995	0.972	0.984

Mantel correlations between the SNP-based GRM and GRM generated by each respective haplotype method with optimal parameter settings are given.

**Table 2:** Number of explanatory variables (haplotype alleles or SNPs) used for genomic prediction.

	FixedHB (window size: 20)	HaploView (GAB)	HaploBlocker (window size: 20, target coverage: 99%)	SNP
DH_KE	84,406	51,860	10,225	369,680
DH_PE	106,620	77,314	14,236	375,204
GC_KE	112,092	72,215	53,178	366,079
GC_PE	127,420	93,445	41,239	392,016

### 3.2 Impact of parameter settings on haplotype construction and prediction accuracy

Haplotype construction was profoundly affected by parameter settings. Therefore, we investigated how selected parameters influenced our success criterion, prediction accuracy. Considering the multitude of parameters and the constrained sample size in this study, our objective was not the exhaustive search for optimal parameter settings in each scenario. Instead, we analyzed the effect of individual parameters on prediction accuracy.

The impact of parameter settings on prediction accuracy of within population prediction (scenario 1) is shown in **Supplementary Figure S2**. With FixedHB, differences in prediction accuracies were negligible when varying window size for the traits under study in the DH populations. In the GC populations, variation in window size affected the five traits differently, with larger windows leading to an increase or decrease of PAs (**Supplementary Figure S2A**). Thus, we chose FixedHB haplotype sets with window size 20 for further comparisons. With HaploView, the three algorithms lead to very similar prediction accuracies, none of them being consistently superior across traits (**Supplementary Figure S2B**). Therefore, we will restrict presentation of results to the default method (GAB) in further comparisons. Regarding HaploBlocker, we observed that haplotype libraries with higher target coverage lead to higher prediction accuracies. For *window size* setting no clear trend was found (**Supplementary Figure S2C**). We chose maximum *target coverage* (99) and default *window size* (20) for further comparisons.

In scenario 2, prediction accuracies of FixedHB decreased for window sizes greater than 20 for most traits (**Supplementary Figure S3A**). With HaploBlocker, the parameter *Min Subgroup* was used to control the minimum number of alleles for each haplotype in each pre-defined subgroup, here DH and GC. Increasing the parameter *Min Subgroup* from 0 to 5, more than halved the length of the haplotypes (**Supplementary Figure S4A**) with only small reductions in genome coverage (**Supplementary Figure S4B**). Based on the general prediction performance of the settings, we identified an optimal value, 40, for *Min Subgroup* for both KE and PE (**Supplementary Figure S4C**), which implied that the best predicting haplotype libraries comprised haplotypes with frequencies higher than ~5% in DH and 8% in GC, respectively. We also tuned the parameter *MCMB* which controls the filtering of haplotypes, with lower *MCMB* resulting in higher genome coverage (**Supplementary Figure S4B**). Lower *MCMB* values were also preferred for prediction, with 1 being the best for KE and 1250 being the best for PE (**Supplementary Figure S4C**).

In scenario 3, window size 10 appeared to be a stable value for FixedHB, although the optimal value varied depending on the specific trait being analyzed (**Supplementary Figure S3B**). Regarding HaploBlocker, preferred settings differed substantially between DH and GC populations. For DH populations, setting *Min Subgroup* > 0 lead to a significant reduction in genome coverage of the haplotype library (**Supplementary Figure S5B**), but to a significant increase in prediction accuracy (**Supplementary Figure S5C**). This indicated an advantage of excluding population specific haplotypes and focusing on haplotypes shared by the two landraces. For GC populations, the impact of *Min Subgroup* on genome coverage (**Supplementary Figure S5B**) and prediction accuracy (**Supplementary Figure S5C**) was not as pronounced. Ultimately, settings with the *Min Subgroup* of 20 for DH and 0 for GC; *MCMC* 1250 for DH and 1 for GC in the *MCMB* were selected as optimal haplotype libraries for comparison (**Supplementary Figure S5C**).

### 3.3 Haplotypes slightly outperformed SNPs in within population prediction

**Figure 3:**
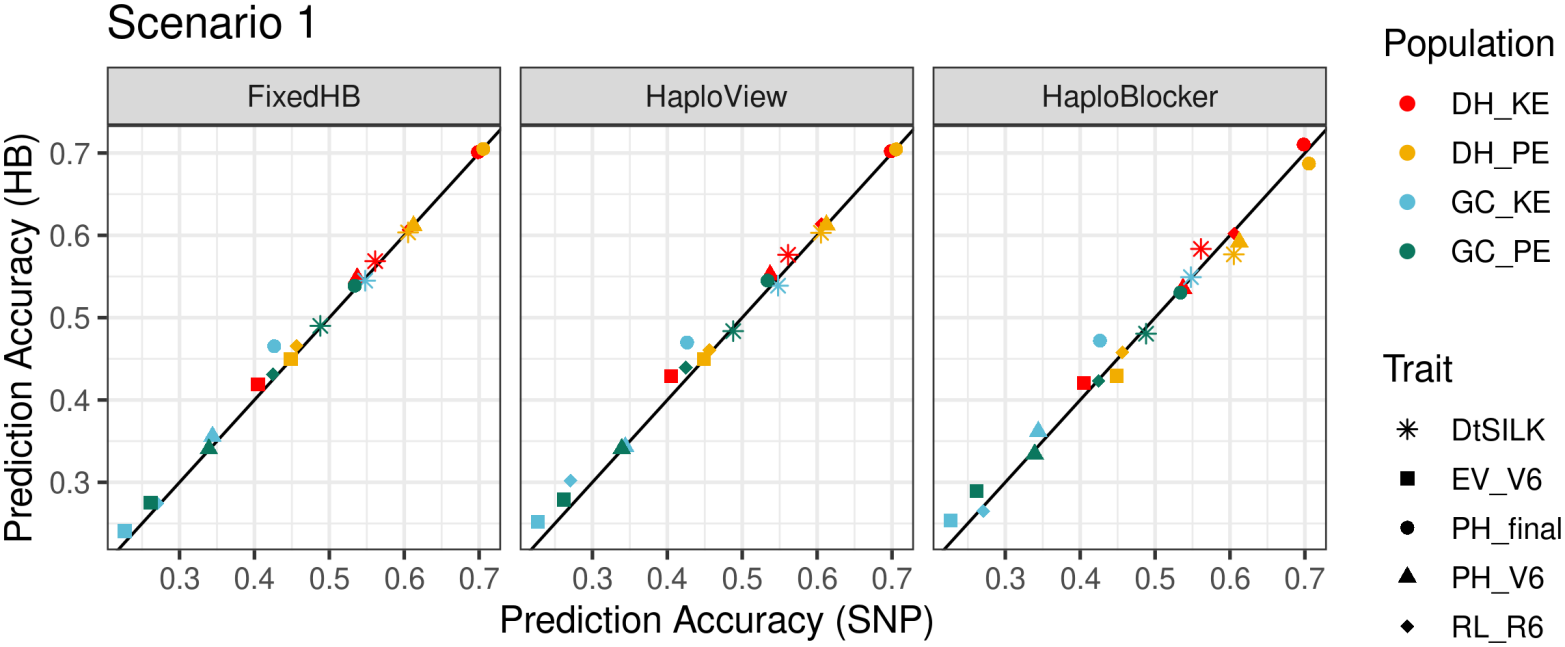
Comparison of accuracies in within population prediction (scenario 1). The x-axis shows the prediction accuracy of SNP-based GBLUP, and the y-axis displays the prediction accuracy of three haplotype-based genomic prediction methods. Colors correspond to the population used as prediction set, different symbols represent five agronomic traits. The haplotype sets were generated using FixedHB with a window size of 20 SNPs, the GAB algorithm for HaploView, and HaploBlocker with a *window size* of 20 and a *target coverage* of 99.

Accuracies for haplotype-based and SNP-based predictions in scenario 1 are shown in **Figure 3** and **Supplementary Figure S6**. FixedHB and HaploView methods yielded similar results. None of the 20 possible trait-population combinations showed a significant decrease in prediction accuracy neither for FixedHB nor for HaploView methods (**Supplementary Table S1**). With FixedHB and HaploView, the highest improvement in prediction accuracy was obtained for final plant height in the GC_KE population (9.1 and 11.6%, respectively). With the HaploBlocker method, none of the tested parameter settings consistently outperformed SNP-based prediction. With *window size* 20 and *target coverage* 99, HaploBlocker showed the highest improvements of all three methods in GC populations (11.8% for final plant height), but it performed below par in four of the five traits in DH_PE.

### 3.4 Focusing on shared haplotypes improves prediction across populations

**Figure 4:**
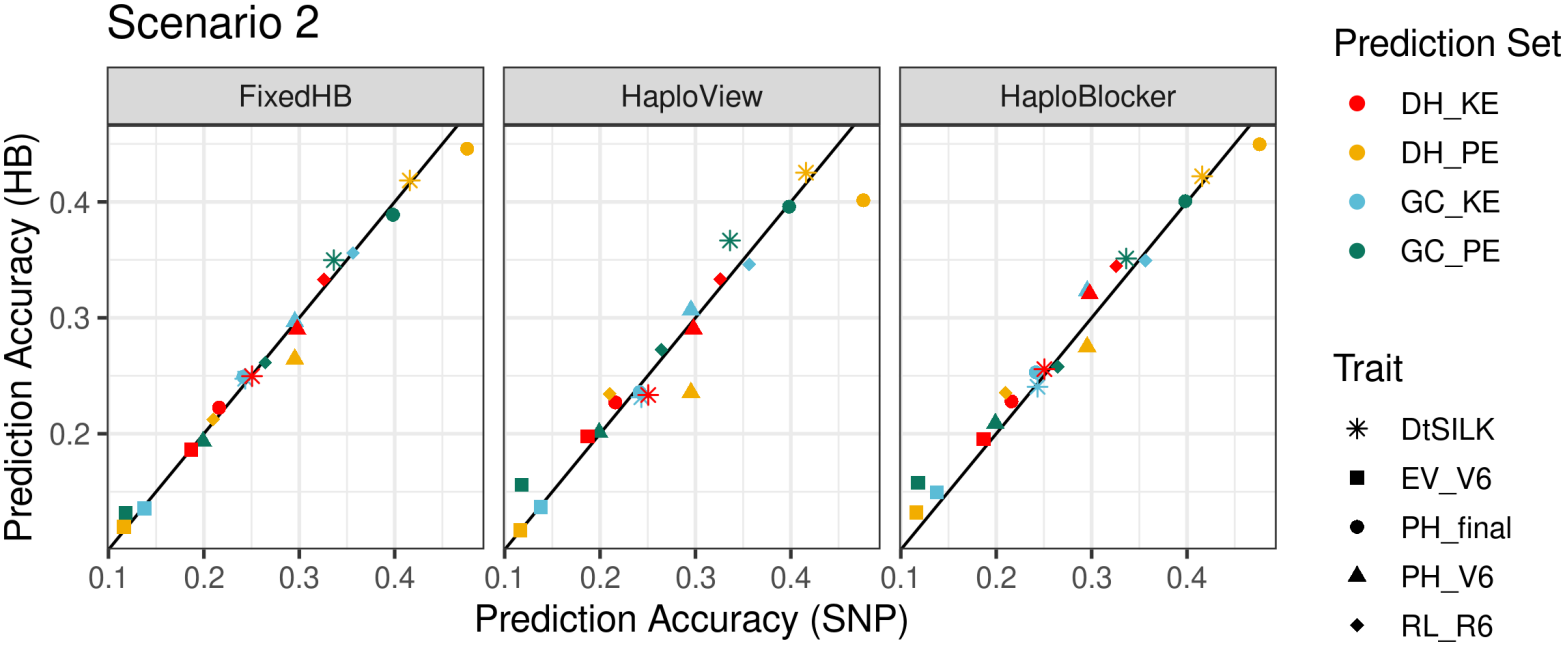
Comparison of accuracies in across population prediction (scenario 2). The x-axis shows the prediction accuracy of SNP-based GBLUP, and the y-axis displays the prediction accuracy of three haplotype-based genomic prediction methods. Colors correspond to the population used as prediction set, different symbols represent five agronomic traits. For FixedHB window size 20 was used; for HaploView the GAB algorithm was used; and for HaploBlocker selected parameter settings were: *MCMB*: 1 for KE and 1250 for PE, *Min Subgroup*: 40 for both landraces.

FixedHB and HaploView based prediction did not show a consistent advantage in scenario 2. Results of HaploView were generally better than the SNP-based method, but for prediction of plant height in DH_PE, accuracies were remarkably inferior (**Figure 4**).

In contrast to the other two methods, it was possible with HaploBlocker to focus on haplotypes shared in pre-defined subgroups, here DH and GC. HaploBlocker haplotype libraries with optimized settings, where population specific haplotypes were excluded, showed improved prediction accuracies compared to SNP-based prediction in most cases. Average improvement was 5%, with a maximum improvement of 33.5% in the best case (early vigor V6 in PE), and a decrease of 6.8% in the worst case (plant height V6 in PE) (**Figure 4**).

### 3.5 Improvement from haplotype-based prediction is trait-dependent in across landrace prediction

**Figure 5:**
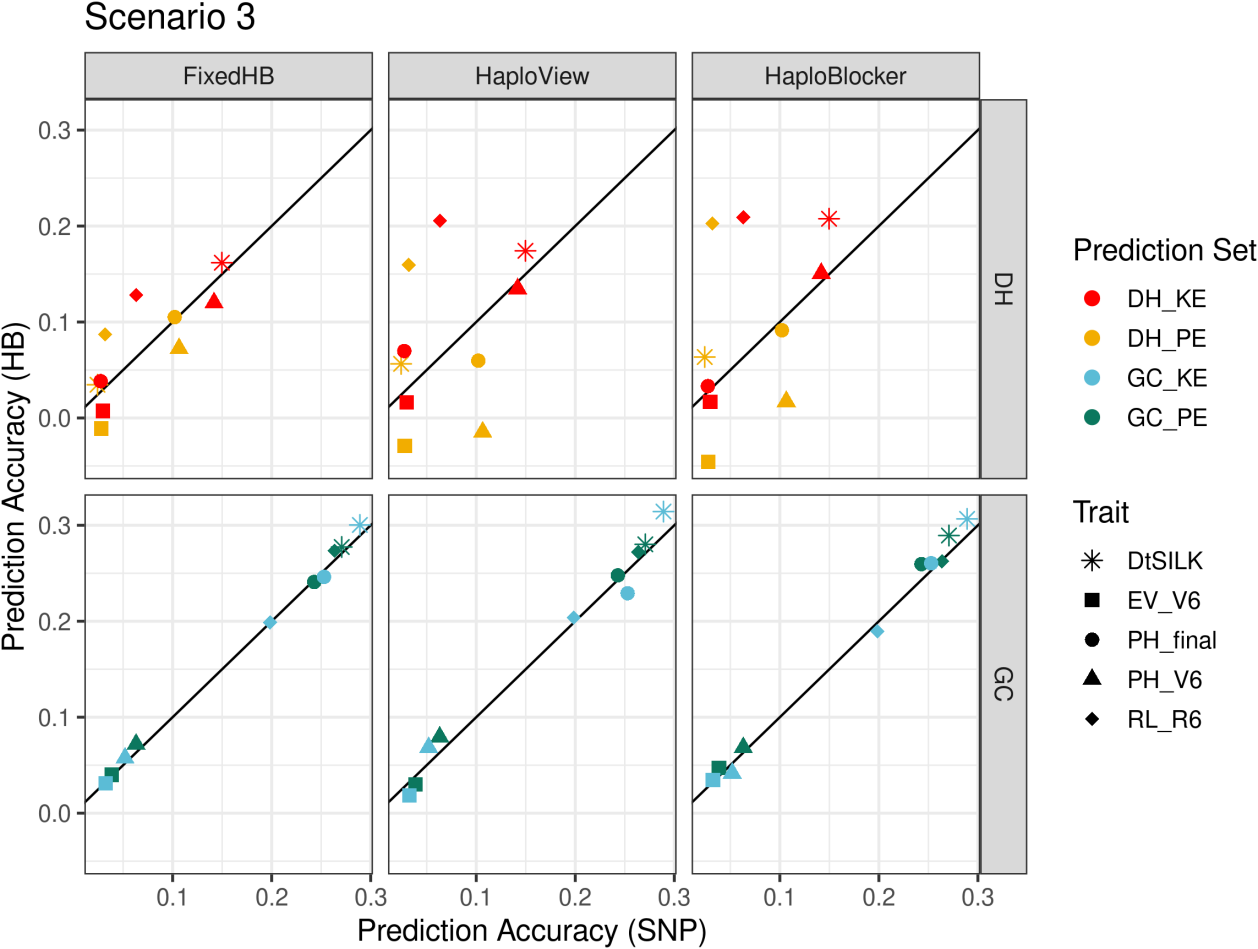
Comparison of accuracies in across landrace prediction (scenario 3), DH at the top and GC at the bottom. The x-axis shows the prediction accuracy of SNP-based GBLUP, and the y-axis displays the prediction accuracy of three haplotype-based genomic prediction methods. Colors correspond to the population used as prediction set, different symbols represent five agronomic traits. For FixedHB window size 10 was used; for HaploView the GAB algorithm was used; and for HaploBlocker selected parameter settings were: *MCMB*: 1250 for DH and 1 for GC, *Min Subgroup*: 20 for DH and 0 for GC.

The prediction results in scenario 3 differed substantially for predictions across landraces in DH populations and in GC populations. In DH populations, both haplotype and SNP-based predictions yielded generally low prediction accuracies (<0.25) (**Figure 5**). However, consistent significant improvements in prediction accuracies were observed for root lodging and female flowering time with all three methods. When predicting in GC populations, prediction accuracies were clearly separated by trait, with the early development traits showing low accuracies. For female flowering time and final plant height, haplotypes somewhat improved the predictions (**Figure 5**).

## 4 Discussion

Using haplotypes instead of individual SNPs can be beneficial for a wide range of applications (Bhat et al., 2021). In plant breeding, haplotypes have been shown to lead to higher accuracies in genomic prediction (Jiang et al., 2018; Sallam et al., 2020; Difabachew et al., 2023) and to be advantageous in the discovery of novel genetic variation in plant genetic resources (Mayer et al., 2020). In the latter case, the construction of haplotypes is obligatory to assess if alleles discovered in genetic resources for traits of interest are already present in elite material or represent novel sources of diversity. However, determining the optimal approach to define haplotypes is not straightforward because the result of haplotype construction methods heavily depend on the genotyping method and density, the linkage disequilibrium in a given species and population, the relatedness of the individuals in that population and haplotype sharing when several populations are analyzed together.

In this study, we explored the suitability of three different haplotype construction methods to capture marker trait associations for quantitative traits. As success criterion we used the accuracy of haplotype-based prediction within and across populations of different genetic structure in comparison to SNP-based prediction. We could show that haplotype-based prediction consistently outperformed SNP-based prediction within and across populations but the choice of the haplotype construction method and the parameter settings determined its success.

### 4.1 Choice of haplotype construction method

FixedHB and HaploView are well suited as haplotype construction methods for maize populations without substructure e.g. scenario 1 in our study. Differences in prediction accuracies were small, but both methods outperformed or performed equally to the SNP-based method across traits, irrespective of the population type (DH or GC) and in both landraces. The landrace with the higher LD (KE) seemed to profit a little more from haplotype-based prediction (**Figure 3**). For the two methods, prediction accuracies were very similar, most likely because both integrate information on LD patterns in the population, HaploView by building blocks based on the LD parameter *D’*, FixedHB by forming haplotypes from adjacent SNPs and leading to haplotypes of comparable length. The advantage of HaploView is that no parameter tuning is necessary and that the total number of haplotype alleles is smaller compared to FixedHB with optimal window size. With HaploView, the total number of haplotypes decreased to 14-24% of the original SNP number. Dimensionality reduction of the genotypic marker matrix was also considered advantageous with LD-based and short-range haplotype methods in other studies (Won et al., 2020; Li et al., 2021; Ye et al., 2022). Depending on the prediction model, the reduction in explanatory variables can also be beneficial for decreasing memory use and computation time, as well as for incorporating interactions between explanatory variables in the statistical model (Vojgani et al., 2021, 2023). When including SNPs that had remained unassigned to haplotypes as additional explanatory variables in the statistical model as proposed by Difabachew et al. (2023), we observed a reduction in prediction accuracy compared to models that did not include them (data not shown). This could be the result of an increase in dimensionality of the model but also of additional noise due to presumably higher genotyping errors of the unassigned SNPs. When deciding on the optimal method to use, it should be noted that with HaploView additional computational work is required compared to FixedHB when new individuals are added to the population, as the LD in the population will change.

HaploBlocker on the other hand did not perform as well in scenario 1 as the other two methods, particularly in the DH populations. There was a striking difference in haplotype length between HaploBlocker and the other two methods in scenario 1 (**Figure 2**). HaploBlocker produced very long haplotype blocks with low frequency in the DH populations especially when target coverage was not at its maximum (**Supplementary Figure S1**). This was a clear disadvantage for prediction (**Figure 3**; **Supplementary Figure S2C**). Our findings are in contrast to the increase in prediction accuracy for haplotypes constructed with HaploBlocker as compared to SNP-based prediction in a wheat diversity panel reported by Difabachew et al. (2023). This discrepancy can very likely be explained by the difference in population structure between the two studies. The wheat diversity panel presumably had a complex IBD structure giving HaploBlocker-based prediction an advantage over SNP-based prediction despite some loss of genome coverage. In the landrace derived maize DH populations, although some IBD patterns exist due to the finite number of landrace S_0_ plants used for DH line generation, the gain in accuracy due to IBD information might not be sufficient to compensate for the loss of information from the reduction in genome coverage.

Due to the strong variation in haplotype lengths generated by HaploBlocker, we modified the genomic relationship matrix in the GBLUP model by weighting each haplotype based on its length on the linkage map, as well as with the number of genes and SNPs it encompasses (see **Supplementary Methods**). Although weighting with the number of genes or SNPs could potentially enhance the accuracy of haplotype-based prediction for certain traits, our findings indicated that these improvements were still insufficient to outperform the SNP-based method (**Supplementary Figure S7**). Consequently, we did not observe any clear advantages resulting from this modification.

In across population prediction (scenarios 2 and 3) none of the three methods outperformed SNP-based prediction consistently. However, with HaploBlocker parameter tuning could be optimized so that specific settings were advantageous in comparison to SNP-based prediction. The most important parameters determining the prediction success of across population and across landrace prediction with HaploBlocker are discussed in the following section.

### 4.2 Choice of HaploBlocker parameter settings for across population prediction

When predicting across populations with the HaploBlocker method, haplotypes should be able to capture IBD genome segments shared by the different populations (Pook et al., 2019). The varying levels of relatedness of the TS and the PS in the three scenarios under study allowed us to investigate how parameters of haplotype construction affected prediction accuracy and how they should be tuned to obtain optimal results.

We investigated the performance of haplotype-based prediction across DH and GC populations derived from the same landrace in scenario 2. The two types of populations differ with respect to the alleles contributed by the capture line, allele frequencies, LD and linkage phase similarities (Hölker et al., 2022). With HaploBlocker, it was possible to build a common haplotype library for DH and GC, with an increasing number of shared haplotypes (parameter *Min Subgroup*) at the expense of genome coverage. Specific parameter settings in HaploBlocker lead to higher prediction accuracy than SNP-based prediction and predictions using the other two haplotype methods (**Figure 4**). The same effect was observed in scenario 3 for DH populations where exclusion of landrace specific haplotypes also led to improved prediction accuracies (**Supplementary Figure S5C**).

In scenario 3, predictions across landraces rely on population-wide LD between markers and QTL in ancestral founders (Habier et al., 2013; Wientjes et al., 2013; Schopp et al., 2017). The low accuracy in prediction across landrace DH populations could be attributed to the low linkage-phase similarities between KE and PE (Hölker et al., 2022). To overcome this, we attempted to increase the level of ancestral LD by increasing the frequency of shared haplotypes, which are assumed to capture IBD information. Despite these efforts, the effectiveness of haplotype-based genomic prediction varied depending on the trait, aligning with findings in similar studies (Won et al., 2020; Weber et al., 2023). This variability might stem from the influence of population-specific QTL, which might play a substantial role in controlling specific traits. In addition, the constructed haplotypes in our study may not capture the IBD segments linked to the QTL that impact these traits comprehensively, leading to the observed discrepancies in prediction accuracy. In prediction across landrace GC populations, haplotypes somewhat improved the accuracy for female flowering time and final plant height, which could potentially be attributed to the shared haplotypes of ancestor FV2 as demonstrated by Hölker et al. (2022).

In scenario 2, we hypothesized that the proportion of haplotypes shared between FV2 and the DH population had an effect on prediction accuracy, with higher values being disadvantageous for prediction, because these haplotypes might be alike in state but have different QTL effects in DH and GC. Thus, the FV2 composition could be an indicator for tuning the HaploBlocker parameter *Min Subgroup* in scenario 2 independent of phenotypic data. Only when setting *Min Subgroup* > 0, HaploBlocker will account for subgroups in the genotypic data and will construct a library of haplotypes shared by both populations. The response of FV2 composition to an increase in *Min Subgroup* showed a convex behavior (**Supplementary Figures S8A, B**). This pattern is likely due to the trade-off between filtering out FV2 exclusive haplotypes and loosing rare haplotypes in the landrace. When choosing the settings with lowest FV2 composition (**Supplementary Figures S8A, B**), 30 for KE and 50 for PE, we arrived at the optimal value (40) chosen based on the success criterion prediction accuracy and the resulting haplotype sets displayed superior or equal performance than the SNP-based prediction for most of the traits (**Supplementary Figure S8C**). These findings emphasize the importance and feasibility of leveraging population background knowledge to establish criteria for optimizing haplotype construction settings.

In this study, our primary focus was on identifying relevant parameters for haplotype construction and comparing the efficiency of different haplotype libraries in genomic prediction in comparison to each other and to SNP-based methods. We could show that haplotypes have the potential to increase prediction accuracies with optimal parameter settings. However, to identify the optimal parameter setting for haplotype construction with broad applicability, it is crucial to tune the parameters in specific training sets accounting for the respective population structure of the prediction sets to ensure the robustness and applicability of the identified parameters across a range of populations and genetic material.

## Data availability statement

Publicly available datasets were analyzed in this study. This data can be found here: https://figshare.com/articles/dataset/Data_from_HoelkerMayer_et_al/17014421 and https://github.com/TUMplantbreeding/HaplotypeGP.

## Author contributions

Y-CL: Conceptualization, Data curation, Formal analysis, Investigation, Methodology, Visualization, Writing – original draft. MM: Conceptualization, Data curation, Methodology, Supervision, Writing – review & editing. DVT: Writing – review & editing, Formal analysis. ToP: Methodology, Writing – review & editing. AH: Data curation, Writing – review & editing. ThP: Resources, Writing – review & editing. MO: Funding acquisition, Writing – review & editing, Resources. C-CS: Conceptualization, Resources, Supervision, Writing – review & editing, Funding acquisition, Methodology.

## References

[B1] Balding D. J. (2006). A tutorial on statistical methods for population association studies. Nat. Rev. Genet. 7, 781–791. doi: 10.1038/nrg1916 16983374

[B2] Barrett J. C. Fry B. Maller J. Daly M. J. (2005). Haploview: analysis and visualization of LD and haplotype maps. Bioinformatics 21, 263–265. doi: 10.1093/bioinformatics/bth457 15297300

[B3] Bhat J. A. Yu D. Y. Bohra A. Ganie S. A. Varshney R. K. (2021). Features and applications of haplotypes in crop breeding. Commun. Biol. 4 (1), 1266. doi: 10.1038/s42003-021-02782-y 34737387 PMC8568931

[B4] Browning B. L. Zhou Y. Browning S. R. (2018). A one-penny imputed genome from next-generation reference panels. Am. J. Hum. Genet. 103, 338–348. doi: 10.1016/j.ajhg.2018.07.015 30100085 PMC6128308

[B5] Cuyabano B. C. D. Su G. S. Lund M. S. (2015). Selection of haplotype variables from a high-density marker map for genomic prediction. Genet. Selection Evol. 47, 1–11. doi: 10.1186/s12711-015-0143-3 PMC4522081 26232271

[B6] Dekkers J. C. M. (2007). Marker-assisted selection for commercial crossbred performance. J. Anim. Sci. 85, 2104–2114. doi: 10.2527/jas.2006-683 17504955

[B7] Difabachew Y. F. Frisch M. Langstroff A. L. Stahl A. Wittkop B. Snowdon R. J.. (2023). Genomic prediction with haplotype blocks in wheat. Front. Plant Sci. 14, 1168547. doi: 10.3389/fpls.2023.1168547 37229104 PMC10203549

[B8] Gabriel S. B. Schaffner S. F. Nguyen H. Moore J. M. Roy J. Blumenstiel B.. (2002). The structure of haplotype blocks in the human genome. Science 296, 2225–2229. doi: 10.1126/science.1069424 12029063

[B9] Gilmour A. Gogel B. Cullis B. Welham S. Thompson R. (2015). ASReml user guide release 4.1 structural specification (Hemel Hempstead, United Kingdom).

[B10] Griffiths A. J. Wessler S. R. Carroll S. B. Doebley J. (2010) Introduction to Genetic Analysis. Ed. Freeman W. H.. (New York City, United States: W.H. Freeman & Co. Ltd).

[B11] Habier D. Fernando R. L. Garrick D. J. (2013). Genomic BLUP decoded: A look into the black box of genomic prediction. Genetics 194, 597. doi: 10.1534/genetics.113.152207 23640517 PMC3697966

[B12] Hess M. Druet T. Hess A. Garrick D. (2017). Fixed-length haplotypes can improve genomic prediction accuracy in an admixed dairy cattle population. Genet. Selection Evol. 49, 1–14. doi: 10.1186/s12711-017-0329-y PMC5494768 28673233

[B13] Hölker A. C. Mayer M. Presterl T. Bauer E. Ouzunova M. Melchinger A. E.. (2022). Theoretical and experimental assessment of genome-based prediction in landraces of allogamous crops. Proc. Natl. Acad. Sci. U. S. A. 119 (18), e2121797119. doi: 10.1073/pnas.2121797119 35486687 PMC9170147

[B14] Hölker A. C. Mayer M. Presterl T. Bolduan T. Bauer E. Ordas B.. (2019). European maize landraces made accessible for plant breeding and genome-based studies. Theor. Appl. Genet. 132, 3333–3345. doi: 10.1007/s00122-019-03428-8 31559526 PMC6820615

[B15] Jiang Y. Schmidt R. H. Reif J. C. (2018). Haplotype-based genome-wide prediction models exploit local epistatic interactions among markers. G3-Genes Genomes Genet. 8, 1687–1699. doi: 10.1534/g3.117.300548 PMC5940160 29549092

[B16] Jiao Y. P. Peluso P. Shi J. H. Liang T. Stitzer M. C. Wang B.. (2017). Improved maize reference genome with single-molecule technologies. Nature 546, 524. doi: 10.1038/nature22971 28605751 PMC7052699

[B17] Li H. W. Zhu B. Xu L. Wang Z. Z. Xu L. Zhou P. N.. (2021). Genomic prediction using LD-based haplotypes inferred from high-density chip and imputed sequence variants in Chinese simmental beef cattle. Front. Genet. 12. doi: 10.3389/fgene.2021.665382 PMC8358323 34394182

[B18] Mantel N. (1967). The detection of disease clustering and a generalized regression approach. Cancer Res. 27, 209–220. 6018555

[B19] Mayer M. Hölker A. C. Gonzalez-Segovia E. Bauer E. Presterl T. Ouzunova M.. (2020). Discovery of beneficial haplotypes for complex traits in maize landraces. Nat. Commun. 11 (1), 4954. doi: 10.1038/s41467-020-18683-3 33009396 PMC7532167

[B20] Meuwissen T. H. E. Hayes B. J. Goddard M. E. (2001). Prediction of total genetic value using genome-wide dense marker maps. Genetics 157, 1819–1829. doi: 10.1093/genetics/157.4.1819 11290733 PMC1461589

[B21] Oksanen J. (2010). Vegan: community ecology package. Available online at: http://vegan.r-forge.r-project.org/.

[B22] Pattaro C. Ruczinski I. Fallin D. M. Parmigiani G. (2008). Haplotype block partitioning as a tool for dimensionality reduction in SNP association studies. BMC Genomics 9, 1–15. doi: 10.1186/1471-2164-9-405 18759977 PMC2547855

[B23] Pook T. Schlather M. de los Campos G. Mayer M. Schoen C. C. Simianer H. (2019). HaploBlocker: creation of subgroup-specific haplotype blocks and libraries. Genetics 212, 1045–1061. doi: 10.1534/genetics.119.302283 31152070 PMC6707469

[B24] R Core Team. (2022). R: A language and environment for statistical computing. (Vienna, Austria). Available at: https://www.R-project.org/.

[B25] Sallam A. H. Conley E. Prakapenka D. Da Y. Anderson J. A. (2020). Improving prediction accuracy using multi-allelic haplotype prediction and training population optimization in wheat. G3-Genes Genomes Genet. Vienna, Austria 10, 2265–2273. doi: 10.1534/g3.120.401165 PMC7341132 32371453

[B26] Schopp P. Muller D. Technow F. Melchinger A. E. (2017). Accuracy of genomic prediction in synthetic populations depending on the number of parents, relatedness, and ancestral linkage disequilibrium. Genetics 205, 441. doi: 10.1534/genetics.116.193243 28049710 PMC5223520

[B27] Unterseer S. Bauer E. Haberer G. Seidel M. Knaak C. Ouzunova M.. (2014). A powerful tool for genome analysis in maize: development and evaluation of the high density 600 k SNP genotyping array. BMC Genomics 15, 1–15. doi: 10.1186/1471-2164-15-823 25266061 PMC4192734

[B28] VanRaden P. M. (2008). Efficient methods to compute genomic predictions. J. Dairy Sci. 91, 4414–4423. doi: 10.3168/jds.2007-0980 18946147

[B29] Vojgani E. Hölker A. C. Mayer M. Schön C.-C. Simianer H. Pook T. (2023). Genomic prediction using information across years with epistatic models and dimension reduction via haplotype blocks. PloS One 18, e0282288. doi: 10.1371/journal.pone.0282288 37000811 PMC10065328

[B30] Vojgani E. Pook T. Martini J. W. R. Holker A. C. Mayer M. Schon C. C.. (2021). Accounting for epistasis improves genomic prediction of phenotypes with univariate and bivariate models across environments. Theor. Appl. Genet. 134, 2913–2930. doi: 10.1007/s00122-021-03868-1 34115154 PMC8354961

[B31] Wang N. Akey J. M. Zhang K. Chakraborty R. Jin L. (2002). Distribution of recombination crossovers and the origin of haplotype blocks: The interplay of population history, recombination, and mutation. Am. J. Hum. Genet. 71, 1227–1234. doi: 10.1086/344398 12384857 PMC385104

[B32] Weber S. E. Frisch M. Snowdon R. J. Voss-Fels K. P. (2023). Haplotype blocks for genomic prediction: a comparative evaluation in multiple crop datasets. Front. Plant Sci. 14. doi: 10.3389/fpls.2023.1217589 PMC10507710 37731980

[B33] Wientjes Y. C. J. Veerkamp R. F. Calus M. P. L. (2013). The effect of linkage disequilibrium and family relationships on the reliability of genomic prediction. Genetics 193, 621. doi: 10.1534/genetics.112.146290 23267052 PMC3567749

[B34] Won S. Park J. E. Son J. H. Lee S. H. Park B. H. Park M.. (2020). Genomic prediction accuracy using haplotypes defined by size and hierarchical clustering based on linkage disequilibrium. Front. Genet. 11. doi: 10.3389/fgene.2020.00134 PMC7067973 32211021

[B35] Ye H. Q. Zhang Z. P. Ren D. Y. Cai X. D. Zhu Q. H. Ding X. D.. (2022). Genomic prediction using LD-based haplotypes in combined pig populations. Front. Genet. 13. doi: 10.3389/fgene.2022.843300 PMC9218795 35754827

