# Supplementary material for: Genomic prediction within and across maize landrace derived populations using haplotypes
Source: Front Plant Sci. 2024 Mar 22;15:1351466. doi: 10.3389/fpls.2024.1351466 (PMC10995330; doi:10.3389/fpls.2024.1351466)
Supplement: Supplementary file 1 [file DataSheet_1.pdf]

Supplementary Material

Genomic prediction within and across maize landrace derived populations using haplotypes

Yan-Cheng Lin[1], Manfred Mayer[1,2], Daniel Valle Torres[3], Torsten Pook[4], Armin C. Hölker[5], Thomas Presterl[5], Milena Ouzunova[5] and Chris-Carolin Schön[1*]

*** Correspondence:** Corresponding Author: chris.schoen@tum.de

1 Supplementary Methods

1.1 Length weighting for HaploBlocker haplotype libraries

Considering the different lengths of haplotype alleles derived from HaploBlocker, we incorporated the haplotype length information by weighting the haplotypes in the haplotype (genomic) relationship matrix ($\mathbf{U}_{\boldsymbol{w}}$) for the GBLUP model. We adapted a formula from (Pook, 2019), to calculate the genomic relationship matrix by assigning weights to haplotypes based on their length information. The formula is as follows:

$$\mathbf{U}_{\boldsymbol{w}} = \frac{\boldsymbol{Z}\boldsymbol{W}^{s}\boldsymbol{Z}'}{2\sum_{h} l_h^s * p_h * (1 - p_h)}$$

In the formula, $\boldsymbol{Z}$ is the n x h haplotype matrix, and p_h is the allele frequency of haplotype h. $\boldsymbol{W}$ is a diagonal matrix with entries l_h indicating the length of each haplotype. In this study, we defined length in three different ways, number of SNPs, number of genes within a haplotype, and length (*cM*) on the consensus linkage map (Haberer et al., 2020). The parameter s is a scaling factor that adjusts the weighting. When s is set to 0, the genomic relationship matrix would be the same as the $\mathbf{U}_{\boldsymbol{H}}$ matrix without weighting. The impact of length weighting was assessed using haplotype set from the DH_PE, with *window size* 20 and *target coverage* 99, which performed the poorest in scenario 1. Scaling factor (s) was set from 0 to 2 with 0.2 increments.

2 Supplementary Figures and Tables

2.1 Supplementary Figures

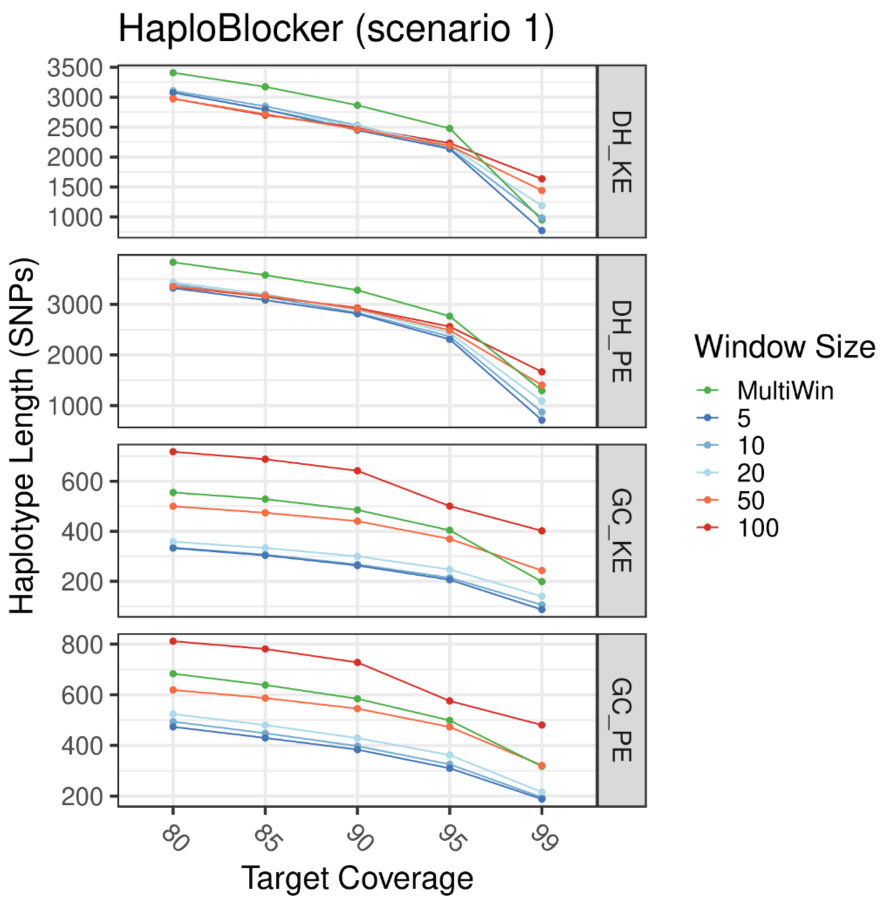

Supplementary Figure 1. Influence of parameters on haplotype length of HaploBlocker in each population of scenario 1. The x-axis is the target coverage setting for haplotype construction, and y-axis displays the average length of the haplotype library. Colors denote the window size used for haplotype construction.

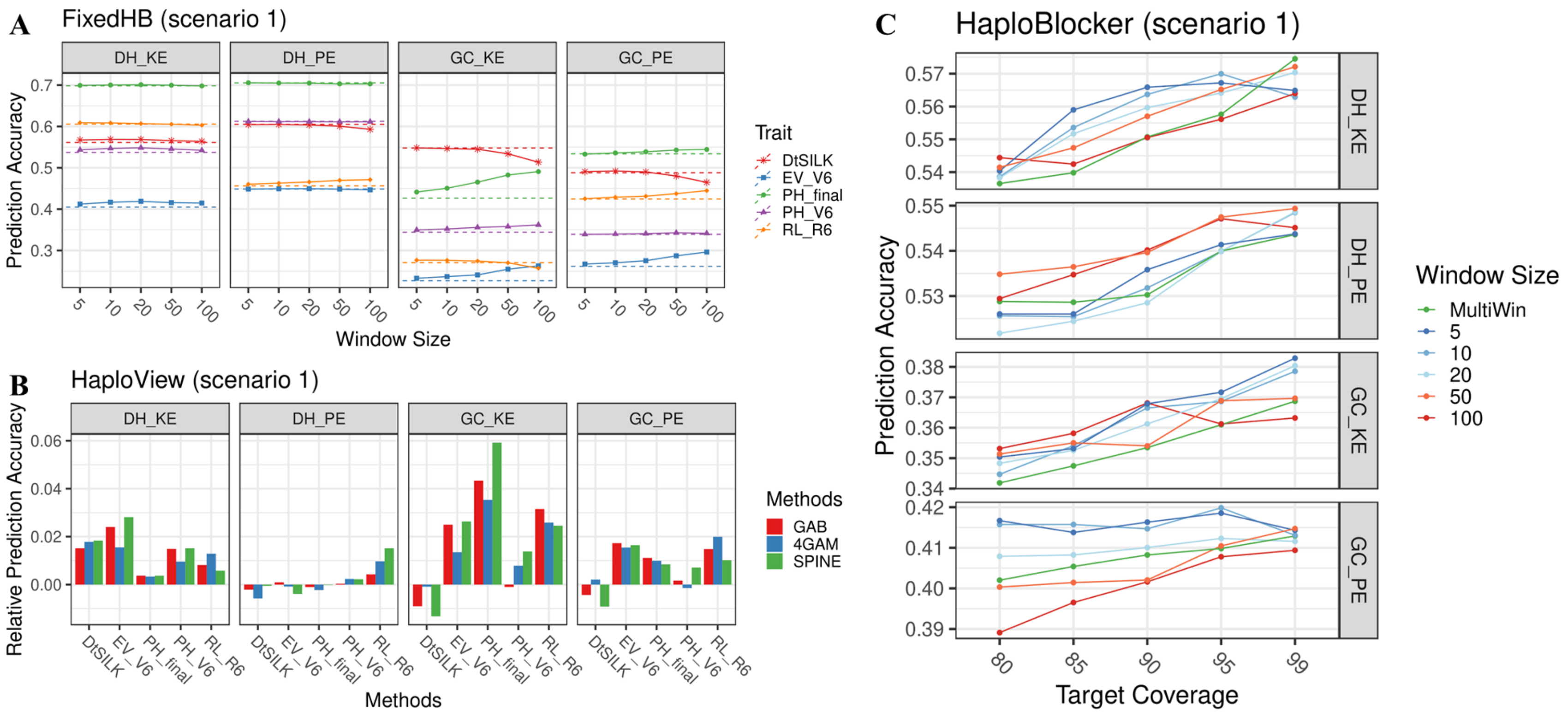

Supplementary Figure 2. Impact of haplotype construction parameters on prediction accuracy in scenario 1. **A:** Influence of FixedHB window size (x-axis) on prediction accuracy (y-axis). Colors of the line represent the traits and the dashed lines indicate the prediction accuracies of the SNP-based method. **B:** Comparison of prediction accuracies between three HaploView algorithms (colors). The x-axis indicates the trait, and the y-axis shows the relative prediction accuracy, with the prediction accuracy of the SNP-based method used as the baseline (0). **C:** Influence of *target coverage* (x-axis) and *window size* (color) settings on prediction accuracy (y-axis) of HaploBlocker. The presented prediction accuracy is the average of the five traits for each haplotype library.

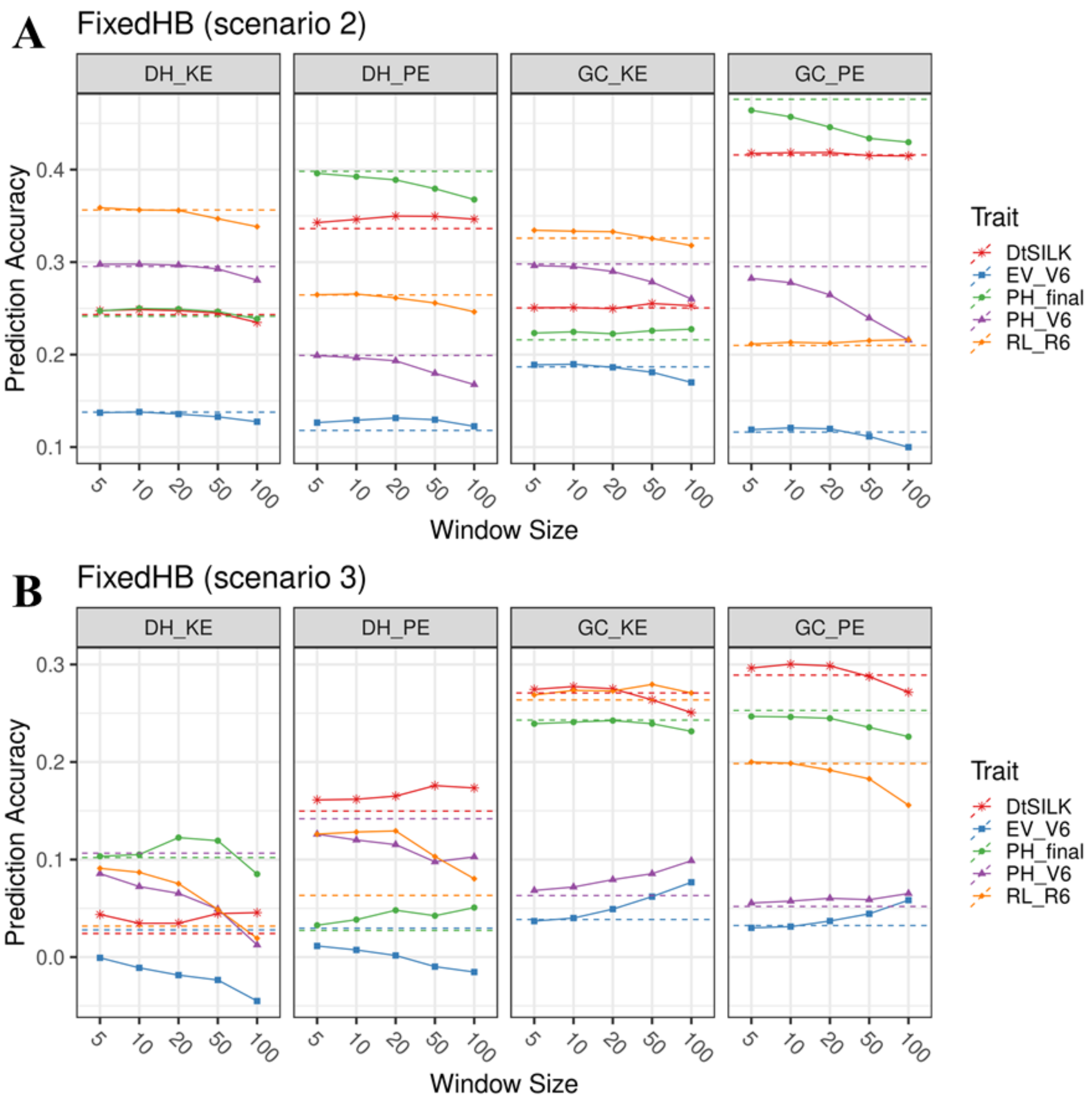

Supplementary Figure 3. Influence of FixedHB window size (x-axis) on prediction accuracy (y-axis) in scenario 2 (**A**) and 3 (**B**). The color of the line represents the trait and the dashed line indicates the prediction accuracy of the SNP-based method.

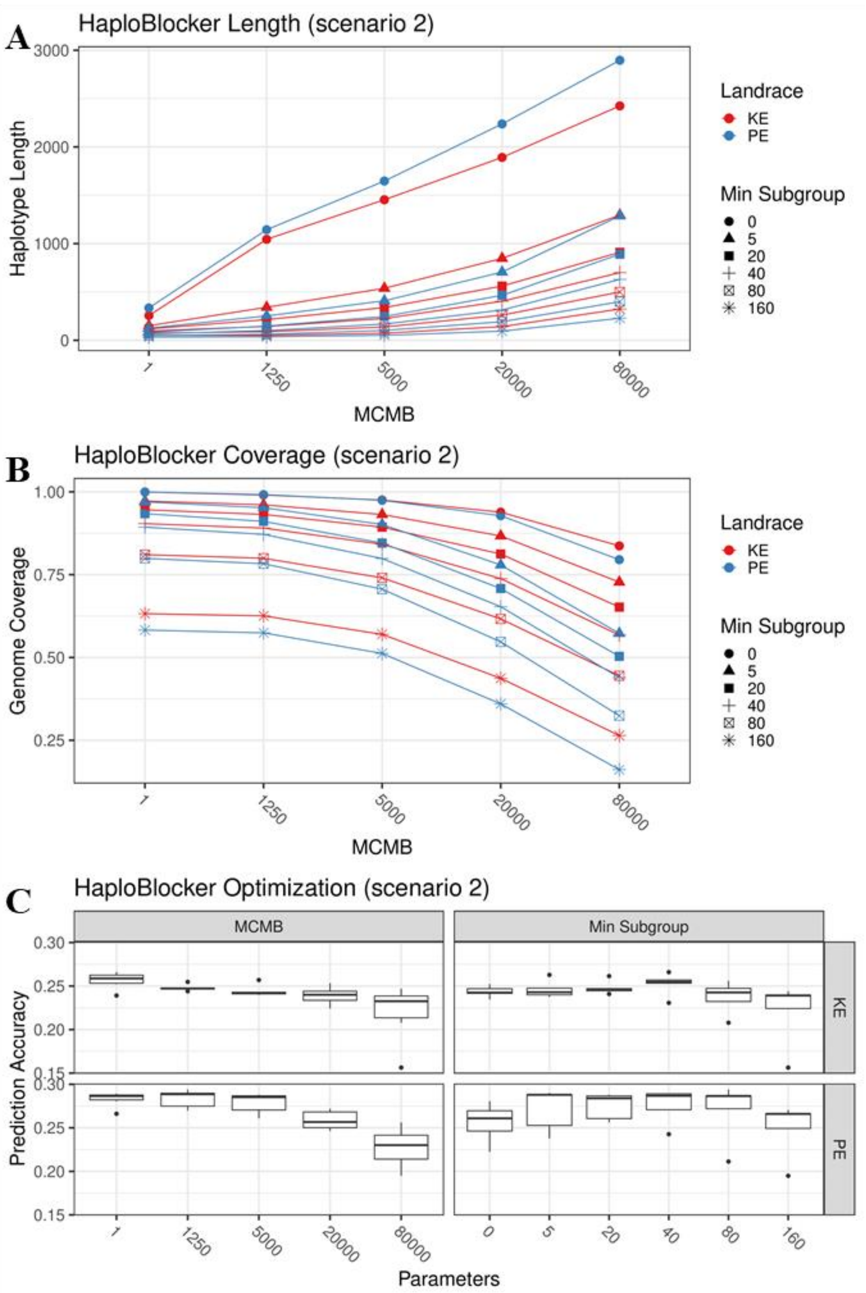

Supplementary Figure 4. Influence of the HaploBlocker parameter configuration on haplotype length (**A**), genome coverage (**B**) and prediction accuracy (**C**) in scenario 2. **A**: The x-axis shows the value of parameter *MCMB*, the symbols denote the parameter *Min Subgroup*, and the y-axis indicates the haplotype length of the respective haplotype library. Colors indicate the landraces used for haplotype construction. **B**: The x-axis shows the value of parameter *MCMB*, the symbols denote the parameter *Min Subgroup* and the y-axis is the genome coverage of the respective haplotype library. Colors indicate the landraces used for haplotype construction. **C**: Box plots depict parameter configurations in relation to prediction accuracy. The x-axis shows the value of parameters, *MCMB* (left) and *Min Subgroup* (right), and the y-axis shows the average prediction accuracy of five traits of the respective haplotype library.

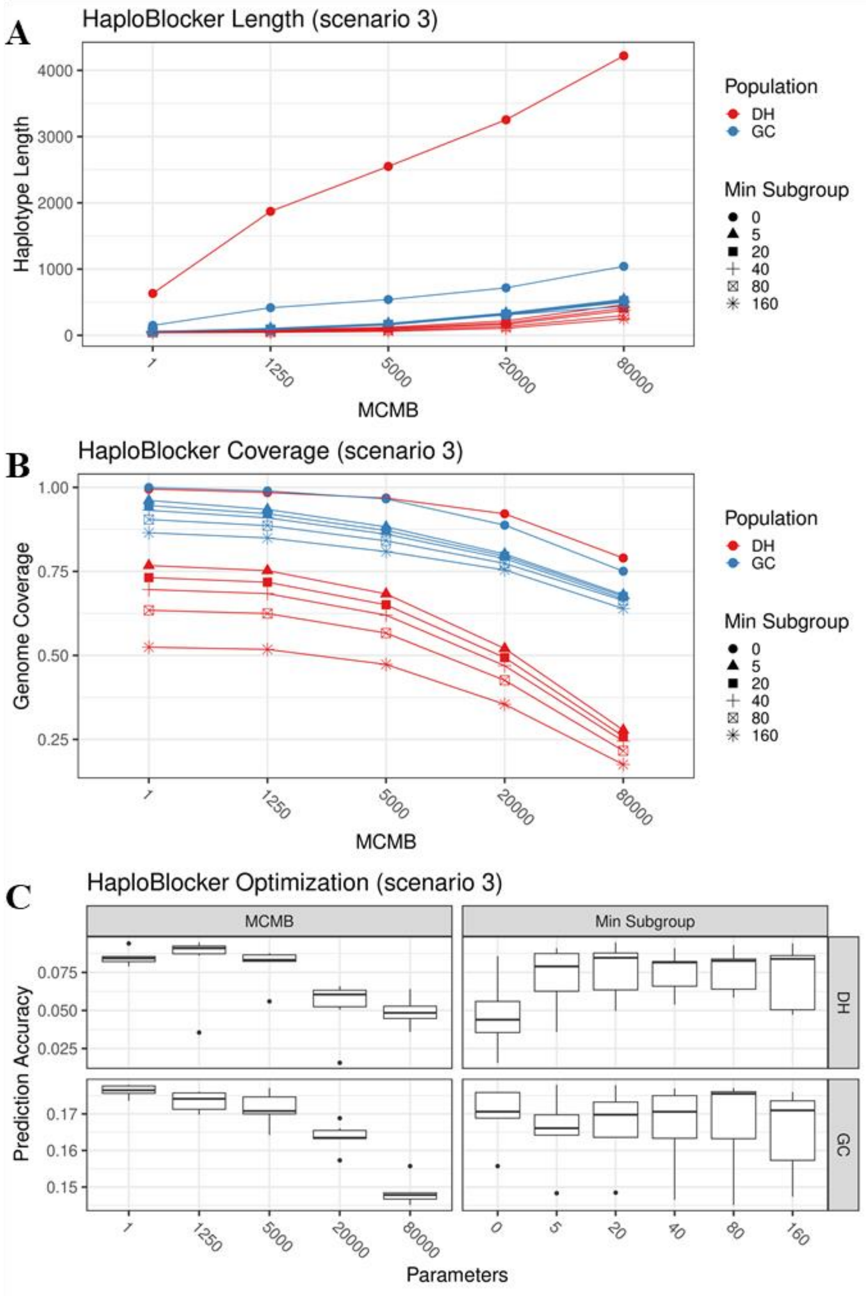

Supplementary Figure 5. Influence of the HaploBlocker parameter configuration on haplotype length (**A**), genome coverage (**B**) and prediction accuracy (**C**) in scenario 3. **A**: The x-axis shows the value of parameter *MCMB*, the symbols denote the parameter *Min Subgroup*, and the y-axis indicates the haplotype length of the respective haplotype library. Colors indicate the populations used for haplotype construction. **B**: The x-axis shows the value of parameter *MCMB*, the symbols denote the parameter *Min Subgroup* and the y-axis is the genome coverage of the respective haplotype library. Colors indicate the populations used for haplotype construction. **C**: Box plots depict parameter configurations in relation to prediction accuracy. The x-axis shows the value of parameters, *MCMB* (left) and *Min Subgroup* (right), and the y-axis shows the average prediction accuracy of five traits of the respective haplotype library.

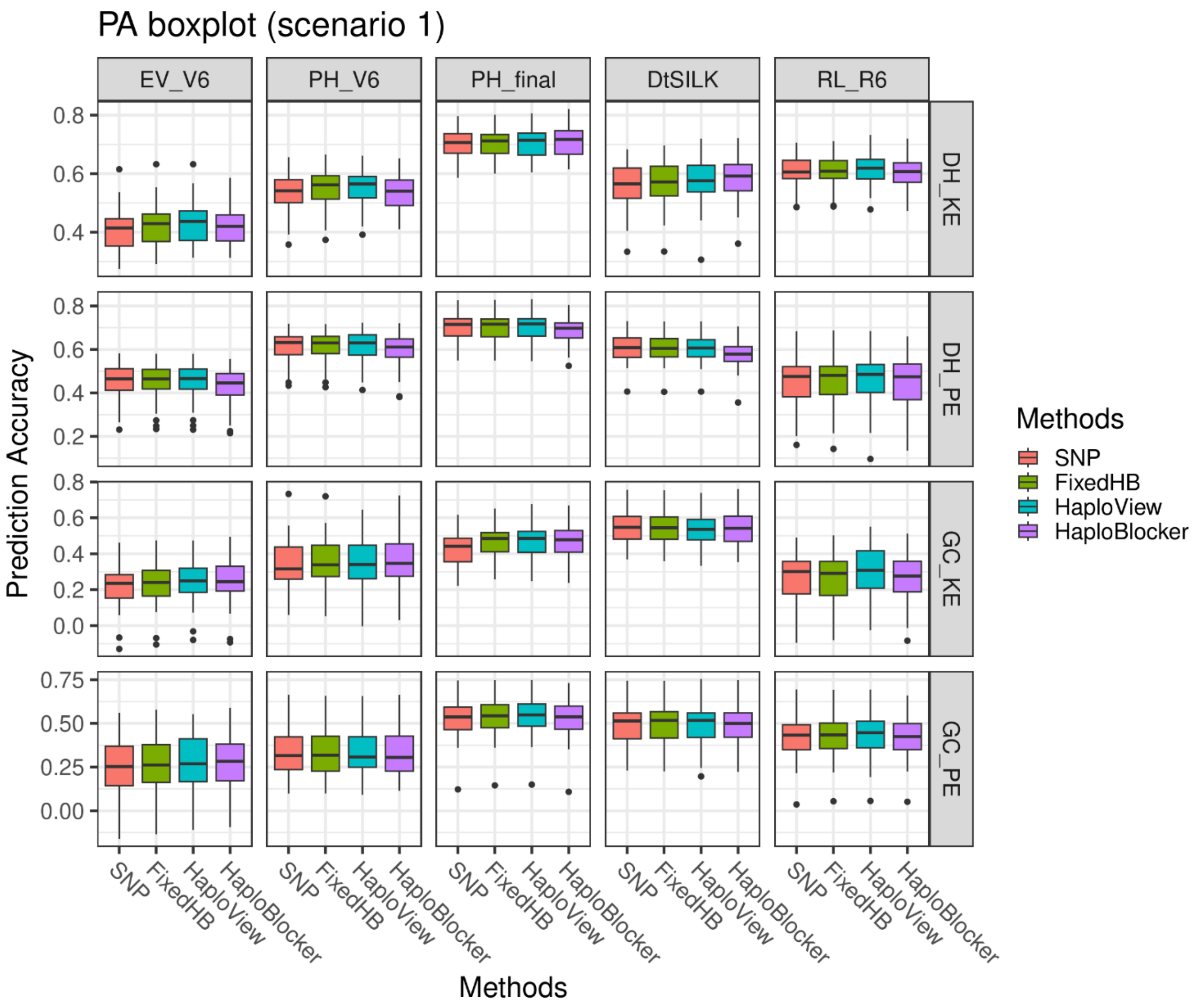

Supplementary Figure 6. Boxplot of prediction accuracies of 50 CV runs in within population prediction (scenario 1). The x-axis and the color indicate the genomic prediction method, the y-axis displays the prediction accuracy. The haplotype sets were generated using FixedHB with a window size of 20 SNPs, the GAB algorithm for HaploView, and HaploBlocker with a window size of 20 and a target coverage of 99.

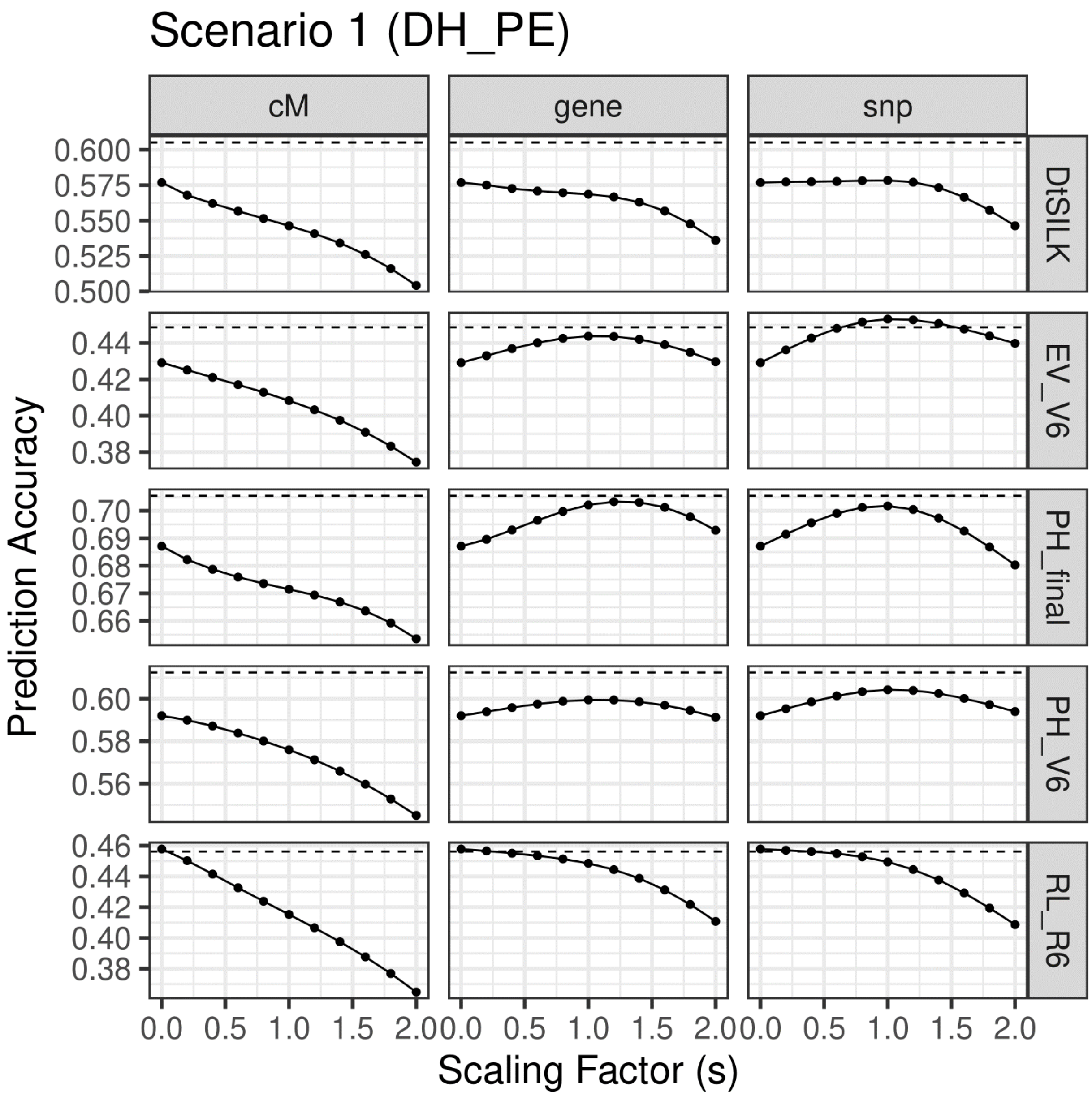

Supplementary Figure 7. Impact of weighting Haploblocker haplotype length on prediction accuracy. Three different haplotype length measures, namely length on linkage map in centimorgan (*cM*), number of genes (gene) and number of SNP (snp), were employed for weighting haplotypes during the construction of genomic relationship matrices. The x-axis represents the weighting scale (*s*), and the y-axis indicates the corresponding prediction accuracy. The dashed line signifies the prediction accuracy achieved by the SNP-based method.

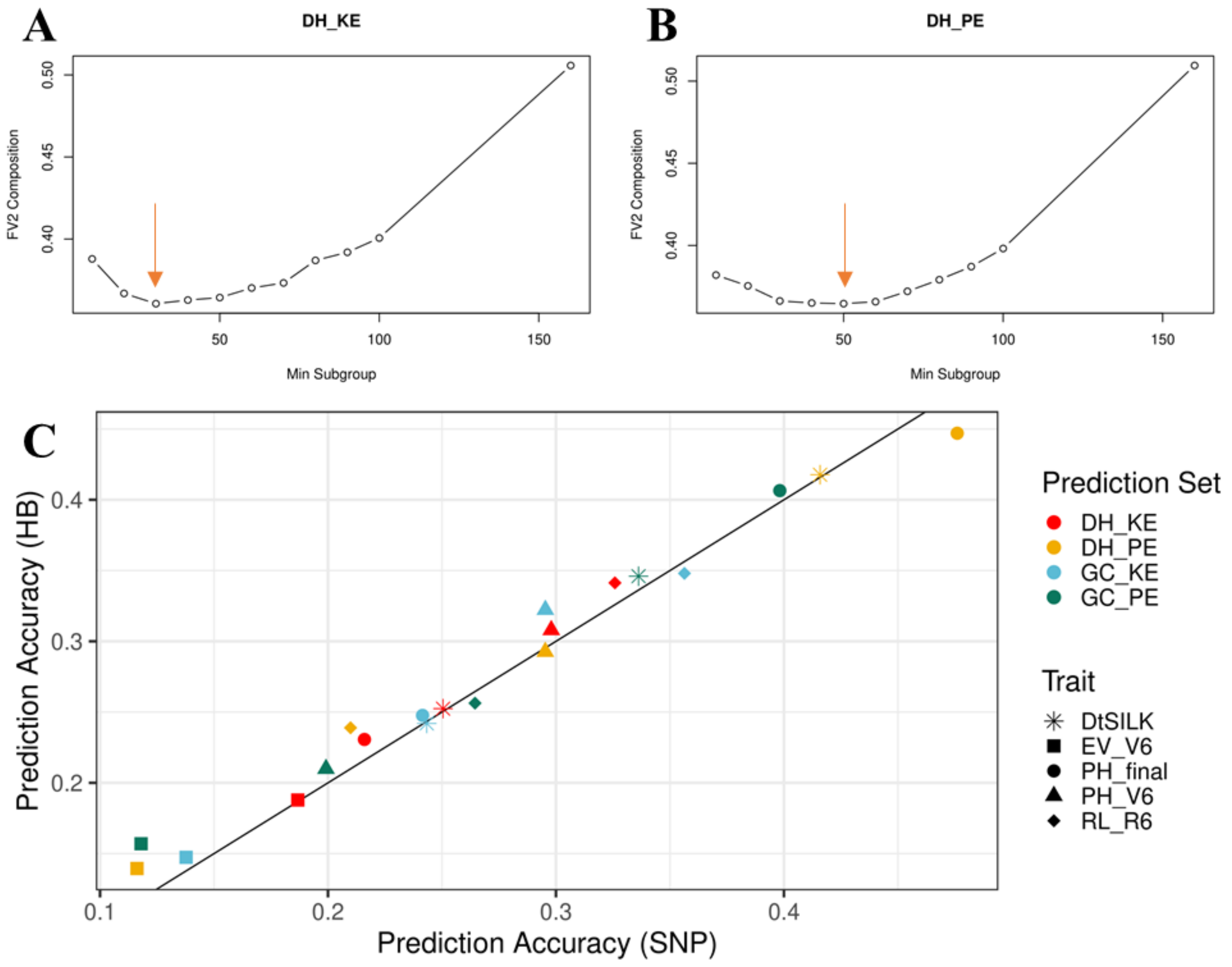

Supplementary Figure 8. Optimization of *Min Subgroup* for HaploBlocker haplotype construction in scenario 2, based on FV2 haplotype composition. (A) For landrace KE and (B) landrace PE, *Min Subgroup* settings were varied from 10 to 100 with a step size of 10, and 160 as the final value. The x-axis represents *Min Subgroup* values, and the y-axis shows the average FV2 composition. The optimal settings were determined as 30 for KE and 50 for PE which minimizing FV2 composition. **C**: We compared the prediction accuracy of SNP-based genomic prediction (x-axis) and haplotype-based (y-axis) using the optimal *Min Subgroup* values based on FV2 composition in scenario 2.

2.2 Supplementary Tables

Supplementary Table 1. Comparison of SNP-based and haplotype-based genomic prediction accuracies in scenario 1, analyzed using Wilcoxon signed-rank test. The 60 trait-population-method combinations were categorized into groups based on whether the prediction accuracy decreased, increased, or remained equal in comparison to the SNP-based method. The test was employed with a significance level of $\alpha = 0.05$, and the Bonferroni correction was applied to account for multiple comparisons.

	Decreased	Increased	Equal
FixedHB	0	10	10
HaploView	0	10	10
HaploBlocker	4	6	10

3 Reference

Haberer, G., Kamal, N., Bauer, E., Gundlach, H., Fischer, I., Seidel, M.A., et al. (2020). European maize genomes highlight intraspecies variation in repeat and gene content. Nature Genetics 52(9), 950-+. doi: 10.1038/s41588-020-0671-9.

Pook, T. (2019). Methods and software to enhance statistical analysis in large scale problems in breeding and quantitative genetics. Georg-August-Universität Göttingen.
